# Open challenges and good experimental practices in the research field of aqueous Zn-ion batteries

**DOI:** 10.1038/s41467-022-28381-x

**Published:** 2022-02-03

**Authors:** Giorgia Zampardi, Fabio La Mantia

**Affiliations:** 1 Universität Bremen, Energiespeicher- und Energiewandlersysteme, Bibliothekstraße 1, 28359 Bremen, Germany; 2 Fraunhofer Institute for Manufacturing Technology and Advanced Materials – IFAM, Wiener Str. 12, 28359 Bremen, Germany

**Keywords:** Batteries, Electrochemistry, Batteries, Materials for energy and catalysis

## Abstract

Aqueous zinc-ion batteries are realistic candidates as stationary storage systems for power-grid applications. However, to accelerate their commercialization, some important challenges must be specifically tackled, and appropriate experimental practices need to be embraced to align the academic research efforts with the realistic industrial working conditions for stationary storage. Within this commentary article, both the open challenges and the good experimental practices are discussed in relation to their impact on the future development of the aqueous Zn-ion technology.

## Abstract (web summary)

Aqueous Zn-based batteries represent a viable and cost-effective technology for electricity grid storage. Here, the authors discuss the most challenging aspects to bridge academic and industrial research and accelerate the adoption of this class of devices on a large scale.

In response to the increasing awareness regarding climate change, many countries have set the goal to increase the share of renewable energy within their total energy production^1^. Therefore, the development of cost-effective and safe energy storage technologies to accumulate the electrical energy harvested from renewable resources before putting it into the power grid has become a critical necessity of our society^2,3^.

In contrast to the already established Li-ion batteries, mild acidic aqueous Zn-ion batteries (ZIBs) operating in a pH range of ca. 4–5.5^4–6^ are excellent candidates as storage systems for power grid applications. The reasons for this lay in their intrinsic high safety and environmentally friendliness, high specific power and high reversibility, non-toxicity, and most importantly, low costs and high abundancy of metallic zinc^4,7,8^. Indeed, the ZIB field has recently received great attention, as evidenced by the exponential growth of related publications in the last 10 years (Fig. 1).

**Fig. 1:**
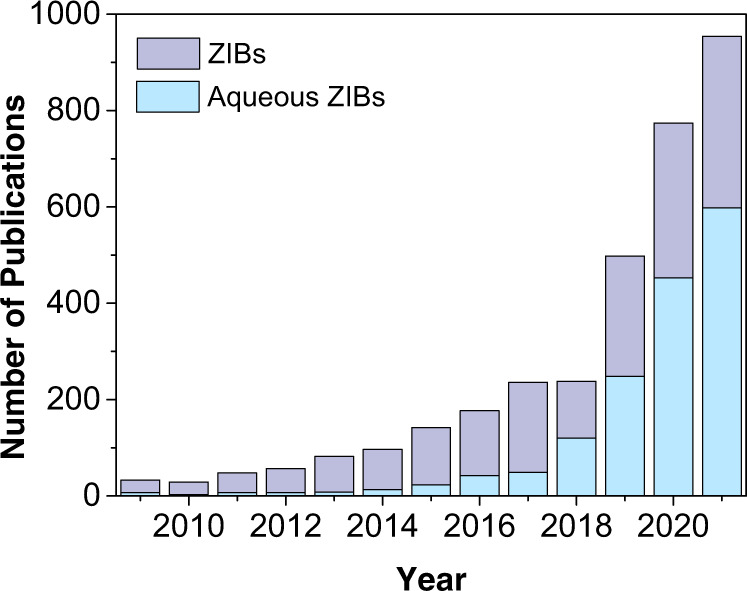
Number of scientific peer-reviewed publications on ZIBs. Total number of scientific publications on ZIBs (organic- plus aqueous-based ZIBs) and specifically on aqueous ZIBs published in recent years (Data collected in November 2021 from Clarivate Web of Science. Keywords used: “zinc-ion batteries”, “aqueous zinc-ion batteries”).

The Zn-ion concept usually consists of a Zn-based negative electrode, onto/from which metallic zinc is electrodeposited and dissolved, and a positive electrode deinserting and inserting Zn^2+^ cations from/within its lattice during the battery cycling^4,8,9^. In mild acidic ZIBs, typical insertion materials for the positive electrodes are based on manganese oxide^10^, vanadium oxides^10^, and Prussian blue analogs (PBAs)^4^.

Although the Zn-ion concept can also be implemented with organic-based electrolytes, a realistic power-grid application of the Zn-ion technology implies the use of water-based electrolyte solutions to ensure high safety levels and keep costs of the final devices at a minimum.

In order to boost the commercialization of aqueous ZIBs as cheap and safe storage devices for the stationary grid, it is worth highlighting the challenges that remain yet to be addressed, together with the adoption of good experimental practices needed to align the academic research efforts with the industrial working conditions envisaged by a practical application of this battery technology.

## Current aqueous ZIBs limitations

### Specific energy and utilization of the zinc anode

Despite the clear advantages that aqueous ZIBs offer in terms of high safety and low costs, the current technology does not attain high enough specific energies (e.g., >40 Wh/kg on full cell scale, including passive elements and casing) that are needed to access the stationary energy market.

The specific energy of a battery system is a function of the specific capacity of both the positive and the negative electrodes (often referred to as cathode and anode, respectively) together with the average discharge voltage of the cell. Although for a proper evaluation of the specific energy of the battery, the inactive components of the cell (such as contacts, current collector, case, etc.) must also be taken into account, Eq. 1 gives an acceptable first estimation of the battery specific energy:

1 $$E=\frac{{Q}_{{cat}}\times {Q}_{{an}}}{{Q}_{{cat}}+{Q}_{{an}}}\times {\triangle V}_{{cell}}$$

where: Q_cat_ Q_an_ are the specific capacities of the active materials constituting the positive and the negative electrodes, respectively, and ΔV_cell_ is the difference of the average operating potentials of the positive and the negative electrode, which defines the average discharge voltage of the cell.

In order to ensure low costs of the final aqueous ZIB, the use of a negative electrode based on metallic zinc is the obvious choice due to the high availability of metallic Zn and its low price (ca. 3.5 $/kg^11^).

**Fig. 2:**
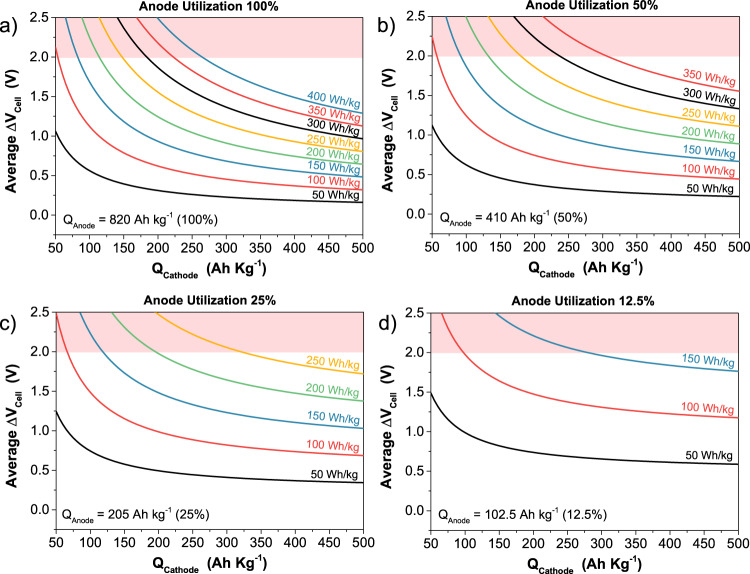
Equi-energy curves of ZIBs. Equi-energy curves of a ZIB calculated from Eq. 1 as a function of the specific charge capacity of a generic cathode. Each graph represents a different extent of utilization of a metallic zinc anode, namely: **a** 100%, **b** 50%, **c** 25%, and **d** 12.5%. Shaded in red is the region in which water splitting occurs, an inaccessible operating regime for ZIBs.

Current academic research efforts to increase the energy content of aqueous Zn-ion batteries generally focus on developing new cathode materials with high specific capacity (e.g., >200 Ah/kg^9^). At the same time, however, the role of the utilization of the zinc-based negative electrode and of its Coulombic efficiency on the electrochemical performance of the battery is widely ignored, despite its heavy influence on the specific energy of a full Zn-ion cell (Eq. 1). The Zn-based anodes routinely employed consist of Zn foils, metallic zinc deposited on a substrate, or composite electrodes with Zn particles within their formulation^7,9^. In order to understand the great challenges that hide behind the Zn-based anode utilization (often referred to as depth of discharge, DOD), it is helpful to consider the equi-energy curves, which are a graphical representation of Eq. 1. Such curves can be calculated as a function of the specific capacity of a generic cathode, as shown in Fig. 2. In each graph, a different utilization (or depth of discharge) of the zinc anode has been taken into consideration. Figure 2a represents the ideal scenario, in which the zinc anode is fully used (100% utilization or depth of discharge) and perfectly matched in terms of charge with the cathode. Figure 2b is the envisaged practical scenario in which the anode is utilized at 50%, meaning that it is two times oversized with respect to the cathode. This means that the total mass of the active material within the electrode formulation (metallic Zn in the case of the anode) is two times more than the one needed to balance the capacity of the cathode. Figure 2c and Fig. 2d represent the more common, worse scenarios in which only 25 and 12.5% of the anode is used, corresponding to four times and eight times oversized anode with respect to the cathode, respectively.

Equation 1 is also helpful when new cathode materials are developed. As it can be seen in its graphical representation in Fig. 2, developing high specific capacity cathode materials (Q_cat_ > 100 Ah/kg) has no practical significance if their operating potential is not high enough to lead to an average discharge voltage of the Zn-ion cell at least higher than 1.0–1.2 V. To make an example, a cathode material having 300 Ah/kg with a working potential of 0.6 V vs Zn/Zn^2+^ tested with a Zn anode utilized at 50% (practical envisaged scenario) would lead to a Zn-ion cell with specific energy lower than 100 Wh/kg (Fig. 2b). Higher specific energy could be reached with a cathode material having only 100 Ah/kg but with a working potential of 1.5 V vs Zn/Zn^2+^, keeping the same utilization of the zinc anode (50%). This effect is evident in the case of the lower zinc anode utilization (Fig. 2d), as the performance of the ZIB is dominated by the Zn anode itself.

Unfortunately, the operational conditions that are mostly used in academic research employ an anode even more than eight times oversized with respect to the cathode, as the positive electrode is usually tested with low active mass loadings of around 1–2 mg/cm^2^ while a metallic Zn foil anode usually has a Zn loading up to 1000 mg/cm^2^^9^.

### Parasitic hydrogen evolution and irreversible Zn-ion losses

Another important challenge that is often ignored within the aqueous ZIB literature concerns the main parasitic reaction occurring within a Zn-ion cell, namely: hydrogen evolution occurring at the zinc-based anode.

The unwanted, although thermodynamically favored H_2_ evolution reaction occurs mainly during the zinc deposition step. Due to the reduction of the hydronium ion H^+^(aq.) to gaseous H_2_, the pH in the proximity of the Zn-based anode locally increases up to values of ca. 6–7.5^12^. In this pH range, the formation and the stabilization of inactive and/or poorly conductive zinc passivation species and clay-like layered double hydroxides are strongly favored^13–17^. This not only causes an irreversible loss of Zn^2+^ from the electrolyte, but also a decrease of the Coulombic efficiency of the Zn anode and thus of the whole Zn-ion cell.

It is worth mentioning that due to the local increase of the pH caused by the occurrence of the H_2_ evolution reaction, the evolution of the morphology of the Zn-based anode during the cycles often shows the appearance of non-homogeneous lamellar deposits, which are typical of the poorly conductive/inactive layered double hydroxides^13–15^, and less commonly of fine dendrites^18^.

## Good experimental practices

### Realistic current rates (C-rates) during the electrochemical tests

The current rate (C-rate) represents the inverse of the time in hours needed by the battery to complete a full discharge or a full charge. It is very important to perform power rate experiments in order to evaluate the efficiency and capacity losses of the electrodes when subjected to different cycling rates, as this represents a realistic working scenario for a battery intended for power grid applications.

However, it is of no practical interest to perform cycle-life duration experiments at C-rates higher than 10 C (meaning full charge or discharge of the battery occurring in 6 min), as the operational currents required by a storage device for the power grid are around 0.5–2 C and do not exceed 10 C^19^. Long cycle life (≥1000 cycles) of the cathode materials is very often claimed in the literature based on C-rates much higher than 10 C, up to ca. 100 C (full charge or discharge of the electrodes in 36 s), where capacitive effects of the double layer often mask the ones related to the Faradic reactions. Moreover, the high current rates strongly underestimate the chemical degradation of the materials, simply because degradation processes depend on time and not on the number of cycles.

However, it is generally known that the higher the C-rate, the slower the aging processes of an insertion material^9,20,21^. In order to collect non-misleading and meaningful results on a realistic estimation of the cycle life of anode and cathode materials, it is critical to choose a 1 C rate for the aging test, as this mimics the rate required for aqueous ZIBs intended to be used as storage devices for the power grid.

### Mass loading of the active materials

The relative mass loading of the active materials for both the anode and the cathode is a very important aspect, which strongly affects both the cycle life of the electrodes and the specific energy of the Zn-ion cell. Despite its clear importance on the cell’s performance, the active mass loading of the electrodes is not generally given proper consideration in the literature.

The electrodes employed for lab-scale measurements often contain a low active material mass loading of ca. 1 mg/cm^2^. When such electrodes are electrochemically tested they experience negligible diffusion limitations and increased electrical connectivity among the different particles, which results in negligible polarization effects. This behavior artificially improves the performance of the material under investigation^9,20^. A realistic evaluation of ZIBs, and in particular of aqueous ZIBs, requires electrodes with an active material mass loading of at least ca. 7–10 mg/cm^2^^9,20^. The mass loading should be high enough that an areal capacity of 2–4 mAh/cm^2^ is reached.

### Electrodes balancing and amount of Zn ions involved during cycling

As already pointed out in the previous section, the relative mass loading of both anode and cathode should be balanced in terms of charge.

As clearly demonstrated by Eq. 1 and Fig. 2, the smaller the mass of the active material within the cathode with respect to the active mass of the anode, the smaller the utilization (depth of discharge) of the anode. Consequently, the more unbalanced the electrodes, the lower the specific energy of the Zn-ion cell. Unfortunately, it is a very common practice to work with cathodes having active material loadings of around hundred-folds smaller than the active mass of the anode (e.g., cathode active material around 1–2 mg/cm^2^, coupled with an anode active mass of ca. 150–300 mg/cm^2^). Unbalanced electrodes are unrealistic as they not only yield artificially high electrochemical performance and Coulombic efficiency of both electrodes (though this effect is more drastic for the Zinc electrodeposition/dissolution process at the anode) but also drastically reduce the specific energy of the Zn-ion cell and vastly increase its costs.

### Reproducibility of the electrochemical tests and data representation

In order to demonstrate the reproducibility of the electrochemical results disclosed in a scientific report or publication, the defining cycling performance characteristics of a material (such as specific capacity, capacity retention, Coulombic efficiency, etc.) should be always reported with mean values and standard deviations averaged over at least three different experiments^21^. In this way, the reproducibility of the acquired measurements would be clearly demonstrated and not only claimed.

Moreover, quantities such as the Coulombic efficiency should be correctly displayed with an appropriate resolution, implying the use of a scale with appropriate dimensions that do not span from 0 to >120%. Only in this way, the data are represented clearly enough to distinguish the possible fluctuation of efficiencies that could be related to important physical phenomena occurring at the electrodes (such as passivation of the surface of the electrode, occurrence of parasitic reactions, degradation of additives in the electrode/electrolyte, etc.).

Last but not the least, the electrochemical analysis of Zn-ion batteries electrodes should be carried out not only in flooded cells containing excess electrolyte (usually around 10 ml, corresponding to ca. 5 ml/cm^2^ with respect to the geometric area of the electrodes), but also in compact ones, where “electrolyte starving” conditions are attained (usually around 50–100 µl/cm^2^ with respect to the geometric area of the electrodes)^9^. During the experiments carried out in cells with electrolyte starving conditions the detrimental effect of the parasitic reactions, leading to the loss of active Zn^2+^ due to the formation of zinc oxide/hydroxide-based inactive compounds, is amplified due to the smaller amount of Zn^2+^-containing electrolyte. This would then result in lower cycle life and lower Coulombic efficiencies with respect to the case where electrolyte flooded conditions are used for the ZIB electrodes analysis^9^.

## Summary and outlook

Due to their promising characteristics, mild aqueous ZIBs represent a viable, green, and cost-effective energy storage technology for stationary grid applications. In order to quickly and efficiently reach this goal, it is worth focusing on the remaining challenges: the increase of the specific energy of the full Zn-ion cell, and the prevention of the parasitic H_2_ evolution reaction occurring during the Zn electrodeposition step.

In order to do so, particular attention must be taken in cycling a fair amount of Zn^2+^ contained within the Zn-based anode with respect to its total mass and in developing cathode materials resulting in an average discharge voltage of the cell at least ≥1.0–1.2 V. Moreover, research efforts should also address the optimization of the electrolyte composition (e.g., additives), towards the development of future water-based electrolytes with the aim to hinder the parasitic hydrogen evolution reaction at the anode.

In order to produce meaningful experimental results, which represent realistic ZIB working conditions, the electrochemical tests should be standardized. In particular: (i) the C-rates employed for the cycle-life assessment of the materials should reflect the need for a stationary power grid (i.e., long-term cycling tests at around 1 C must be carried out); (ii) the electrodes employed during the electrochemical tests should have a realistic amount of active material mass loading (e.g., in the 7–15 mg/cm^2^ range); (iii) both electrodes (anode and cathode) of the electrochemical cell should be balanced in terms of charge. Moreover, attention should be paid to testing Zn-ion battery electrodes and full cells not only in flooded conditions but also in cells where “electrolyte starving” conditions are attained, as this would mimic the real working conditions of the battery.

On the other side, in order to demonstrate the robustness and reproducibility of the research findings, experimental data (such as Coulombic efficiency, specific capacity, specific capacity retention, etc.) should always be averaged over at least three different measurements.

Only through focusing on the main issues that are currently limiting the practical application of aqueous ZIBs and the adoption of good experimental practice in the academic research, which reflects the real working conditions envisaged for stationary power grid applications, researchers will be able to push forward the development of the aqueous Zn-ion technology, allowing it to permeate the stationary energy market.
